# Laparoscopic Cholecystectomy for Acute Calcular Cholecystitis in a Patient with Ventriculoperitoneal Shunt: A Case Report and Literature Review

**DOI:** 10.1155/2015/845613

**Published:** 2015-12-22

**Authors:** Abdullah A. Albarrak, Sami Khairy, Alzahrani Mohammed Ahmed

**Affiliations:** ^1^Surgery Department, College of Medicine, Al Majmaah University, P.O. Box 47212, Riyadh 11552, Saudi Arabia; ^2^Division of Neurosurgery, Department of Surgery, King Abdulaziz Medical City, P.O. Box 22490, Riyadh 11426, Saudi Arabia; ^3^Division of General Surgery, Department of Surgery, King Abdulaziz Medical City, P.O Box 22490, Riyadh 11426, Saudi Arabia

## Abstract

Management of patients who have ventriculoperitoneal shunt presenting with acute calcular cholecystitis has remained a clinical challenge. In this paper, the hospital course and the follow-up of a patient presenting with acute calcular cholecystitis and ventriculoperitoneal shunt managed with laparoscopic cholecystectomy are presented followed by literature review on the management of acute calcular cholecystitis in patients who have ventriculoperitoneal shunts.

## 1. Introduction

Gallbladder stones represent a common pathology in the population, estimated to be 6–9% in the population [1]. Laparoscopic cholecystectomy is currently the standard of care for treating patients with symptomatic gallstones if they have acceptable fitness for surgery. However, patients who have ventriculoperitoneal (VP) shunts represent a special group of patients who require special attention. In this paper, we present a case report of a patient presenting with acute calcular cholecystitis and in situ VP shunt followed by literature review. 

## 2. Clinical Presentation

41-year-old female came to the emergency department with a complaint of significant right upper quadrant abdominal pain for 3 days, constant and radiating to the tip of the right scapula. The pain was associated with nausea and few bouts of vomiting. There was no history of jaundice and no history of fever at the time of admission. The systematic review of symptoms was unremarkable otherwise. The patient had a medical history of diabetes, resolved deep vein thrombosis and pulmonary embolism, and completed treatment three years prior to this presentation. The patient had a history of pseudotumor cerebri for which a VP shunt was inserted 3 years ago.

On examination upon presentation, the patient was conscious, alert, and oriented. She was in pain and mildly dehydrated. She had normal vitals and she was not jaundiced. The abdominal exam showed the scar of the VP shunt in the right upper quadrant. It was soft and lax with significant tenderness in the right upper quadrant with positive Murphy's sign.

Laboratory results are as follows: white cell count, 13.7 trillion cells/L, normal (total bilirubin, direct bilirubin, serum amylase, urea and electrolytes, and coagulation profile). Pregnancy test was negative.

**Figure 1:**
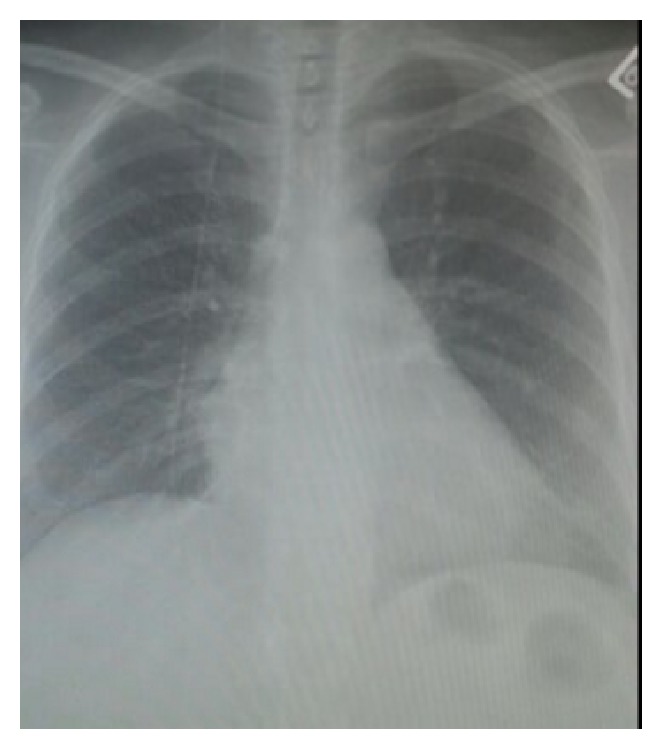
Erect chest X-ray.

**Figure 2:**
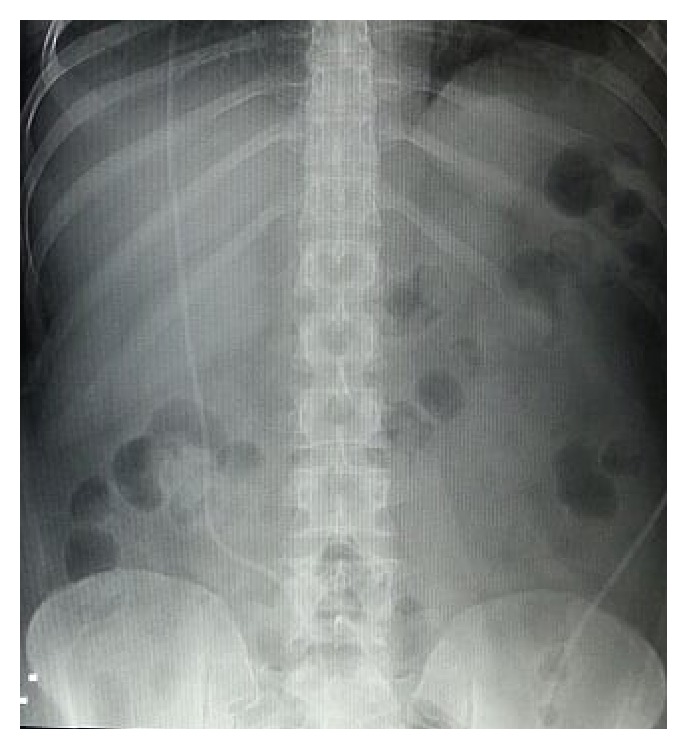
Supine abdominal X-ray.

**Figure 3:**
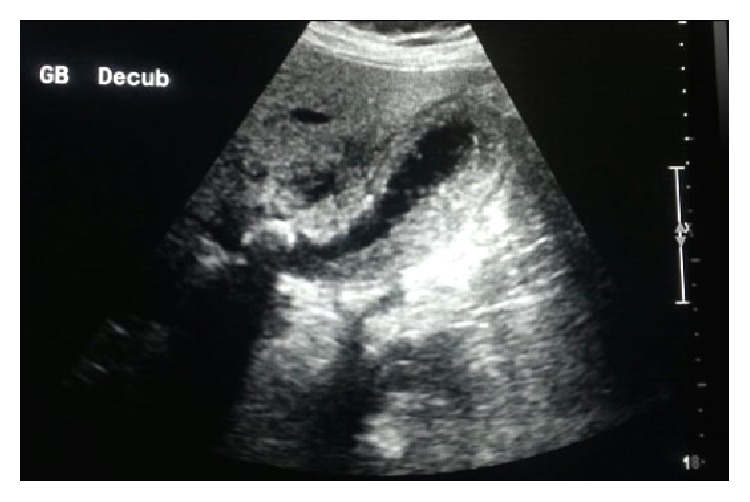
Gallbladder ultrasound.

Chest and abdominal X-rays showed the VP shunt with no other signs seen, Figures 1 and 2. Ultrasound was done which showed impacted stone and the neck of the gallbladder, distended gallbladder with wall edema and double wall sign. Pericholecystic fluid and positive sonographic Murphy's sign were observed, Figure 3.

The patient was admitted, rehydrated, kept nil per os, and started on piperacillin and tazobactam and subcutaneous insulin sliding scale.

On the next day, the patient complained of increase in the abdominal pain. The patient spiked fever reaching up to 39.2 degrees Celsius, whereas the other vital signs were normal. The patient showed increased tenderness in the right upper quadrant. The white cell count was 25 trillion cells/liter, and the patient was taken urgently to the operating room for emergency cholecystectomy as she clinically and biochemically worsened despite IV antibiotic therapy.

In the operating room, the patient was placed in supine position with pneumatic compression device applied. Conventional four-port laparoscopic cholecystectomy was carried out. The pneumoperitoneum was maintained at 12 mmHg. The intraperitoneal part of the VP shunt was moved into the pelvis away from the operative field. The gallbladder was distended with gangrenous fundus surrounded by the omentum. The omental flimsy adhesions were bluntly released. The fundus of the gallbladder was aspirated with a special laparoscopic aspiration needle; then, the fundus was held for retraction. The attention was moved then to the hepatocystic triangle. The cystic duct and artery were clipped after attaining the view of safety. Then, the gallbladder was released from its bed and extracted through the epigastric port using the Endo-Catch. There was a minimal spillage during the operation. The surgical bed was examined; there was no bleeding or bile leak. Irrigation and suctioning were done. Closed suction drain was inserted in Morison's pouch. The fascia was closed with Vicryl 0 for the epigastric and the supraumbilical ports. The skin was closed using skin clips.

The postoperative period showed a dramatic improvement of her abdominal pain, there were no more spikes of fever, and the white cell count went back to normal the second day postoperatively. The drain output was serous; hence, it was removed on the second day postoperatively. The patient was discharged on the third day postoperatively. She was kept on piperacillin and tazobactam until the discharge. The patient has never complained of any neurological symptoms. Of note, neurosurgical consultation was initiated to weigh any extra operative steps with regard to the intraperitoneal component of the VP shunt.

Her first postoperative visit demonstrated uneventful recovery. Last follow-up was three months postoperatively and she continued to do well.

The histopathology showed severe necrotizing acute calcular cholecystitis.

## 3. Discussion

VP shunt is the main surgical intervention for the patients who have hydrocephalus. VP shunts are made of silicon tube which is placed subcutaneously, connecting the brain's lateral ventricle to the peritoneum [2, 3].

Early laparoscopic cholecystectomy is considered safe and recommended for patients presenting with acute calcular cholecystitis, unless otherwise contraindicated [4].

The observation of high intracranial pressure (ICP) in animal models has raised the concern about the laparoscopic safety profile [5]. However, this complication has never been observed in several case reports which were published afterward even with ICP monitoring in some cases [6–11]. Secondly, Neale and Falk [12] studied in vitro the tolerance of nine different types of VP shunts which showed that seven shunts developed seal leak at minimum of 80 mmHg, a level which is severalfold above the pressure maintained during laparoscopic surgery.

The laparoscopic surgery in patients who have VP shunt has been widely discussed specially in urology and gynecology procedures.

On the other hand, for acute laparoscopic cholecystectomy there are only few studies. The biggest study is a chart review of 23 cases that had laparoscopic cholecystectomy during the period 1994–2003 in United States retrieved from the Veterans Affairs databases [13]. All the patients in this study did not have a congenital cause of their hydrocephalus; they were mostly males (92%). Eight of their patients had acute calcular cholecystitis. The timing of cholecystectomy was not mentioned, that is, early or late. It was found that the rate of conversion from laparoscopic to open cholecystectomy was 57% which was attributed to dense adhesion. Two patients required shunt removal and replacement secondary to postoperative shunt infection. Interestingly, those two patients did not receive prophylactic antibiotics perioperatively. Two patients had their shunts temporarily exteriorized postoperatively and one patient had shunt repositioning preoperatively into the left upper quadrant of the abdomen and five surgeons used packing of the shunt with simple gauzes away from the operative field [12].

Collure et al. [6] reported the use of laparoscopy for four cases who developed acute calcular cholecystitis in patients who had VP shunt for more than 1 year. One out of the four patients had a conversion to open procedure secondary to the extensive inflammation. All the patients received prophylactic antibiotic preoperatively and then every eight hours for nonreported duration. Their postoperative course was unremarkable in regard to shunt infection or neurological complications.

Martínez-Lage et al. reported the laparoscopic cholecystectomy for a patient who presented with acute calcular cholecystitis with prior VP shunt. No ICP monitoring was used and the patient had unremarkable postoperative course [14].

For our patient, she received intravenous antibiotics in form of piperacillin/tazobactam pre- and postoperatively for 3 days until the discharge. No ICP monitoring was used. The patient's postoperative course was unremarkable for any neurological complications or shunt infections.

## 4. Conclusion

In patients with VP shunts, emergency laparoscopic cholecystectomy for acute cholecystitis seems to be a safe approach. Intraperitoneal component of the VP shunt does not seem to necessarily increase the risk of intra-abdominal or central nervous system infections. ICP monitoring is not particularly required. It is recommended, however, that the procedure is performed by an experienced laparoscopic surgeon in this particular cohort of patients in order to minimize the chance of spillage and contamination. Also, a neurosurgical consultation before and after the laparoscopic procedure is recommended. An extended course of antibiotics is suggested.
